# Comparison of pulmonary lesions using lung ultrasound and high-resolution computed tomography in adult patients with primary humoral immunodeficiencies

**DOI:** 10.3389/fimmu.2022.1031258

**Published:** 2022-10-25

**Authors:** Marcin Ziętkiewicz, Natalia Buda, Ewa Więsik-Szewczyk, Maciej Piskunowicz, Dominika Grzegowska, Karina Jahnz-Różyk, Zbigniew Zdrojewski

**Affiliations:** ^1^ Department of Rheumatology, Clinical Immunology, Geriatrics and Internal Medicine, Medical University of Gdańsk, Gdańsk, Poland; ^2^ Department of Internal Medicine, Pneumonology, Allergology and Clinical Immunology, Central Clinical Hospital of the Ministry of National Defense, Military Institute of Medicine, Warsaw, Poland; ^3^ Department of Radiology, Medical University of Gdansk, Gdańsk, Poland; ^4^ Care and Treatment Facility, University Clinical Center, Gdańsk, Poland

**Keywords:** Lung ultrasonography, high-resolution computed tomography, chest sonography, antibody deficiencies, immunodeficiency, pulmonary fibrosis, interstitial lung disease

## Abstract

Pulmonary involvement is the most common complication in patients with predominantly antibody deficiencies (PADs). Therefore, patients require repeated imaging tests. Unlike high-resolution computed tomography (HRCT), lung ultrasonography (LUS) does not expose patients to X-rays or contrast agents, and can be performed even at the bedside. This study aimed to evaluate lung lesions using simultaneous LUS and HRCT in a group of patients with PADs. Twenty-nine adult patients (13 women and 16 men) diagnosed with PADs according to the ESID criteria (23 Common variable immunodeficiency, 2 X-linked agammaglobulinemia, 2 IgG subclass deficiencies, and 2 Unspecified hypogammaglobulinemia) were included in the study. The mean age was 39.0 ± 11.9 years. The mean time elapsed between the first symptoms of PADs and the examination was 15.4 ± 10.1 years. Lung ultrasonography and high-resolution computed tomography were performed simultaneously according to a defined protocol during the clinic visits. In both examinations, lesions were compared in the same 12 regions: for each lung in the upper, middle, and lower parts, separately, front and back. A total of 435 lesions were described on LUS, whereas 209 lesions were described on HRCT. The frequencies of lesions in the lung regions were similar between LUS and HRCT. In both examinations, lesions in the lower parts of the lungs were most often reported (LUS 60.9% vs. HRCT 55.5%) and least often in the upper parts of the lungs (LUS 12.7% vs. HRCT 12.0%). The most frequently described lesions were LUS consolidations (99; 22.8%) and HRCT fibrosis (74; 16.5%). A statistically significant relationship was found in the detection of fibrosis in 11 of the 12 regions (phi = 0.4−1.0). Maximum values of the phi coefficient for the upper part of the left lung were recorded. Compared with HRCT, LUS is an effective alternative for evaluating and monitoring pulmonary lesions in adult patients with PADs, especially for pulmonary fibrosis.

## Introduction

According to the classification developed in 2019, the group of diseases defined as inborn errors of immunity (IEIs) includes over 400 entities (1). However, it should be emphasized that new diseases are described every year. IEIs represent a heterogeneous group of diseases with significantly different clinical presentations. The epidemiology of IEI is challenging to estimate, but in most registries, at least half of the cases are classified as predominantly antibody deficiencies (PADs) (2). Among others, this group includes diseases such as common variable immunodeficiency (CVID), X-linked agammaglobulinemia (XLA), or immunoglobulin G subclass deficiency.

Pulmonary complications are estimated to affect about 60% of patients with PADs and up to 90% patients with CVID (3). Recurrent bacterial respiratory infections are the leading symptoms in this patient group (4) and are often the main reason for the expansion of the diagnosis of primary immunodeficiencies. Frequent or severe respiratory infections can cause structural lung damage that may promote chronic pulmonary diseases such as bronchiectasis, atelectasis, or fibrosis. Early diagnosis and management with prophylactic antibiotics and Ig replacement therapy reduce the frequency of infections and their long-term effects (5).

Many patients with PAD also have pulmonary non-infectious complications such as the previously mentioned bronchiectasis or fibrosis, as well as asthma, interstitial disease, or malignancy. Depending on the population studied, the incidence of complications is various. It is estimated that asthma occurs in 31.2% of patients with CVID and 10.3% of patients with XLA (6). Bronchiectasis occurs in 25 to 79% of patients with PADs (7–9). Less common is interstitial lung disease (ILD), described mainly in patients with CVID (10-20%) (8). A particular form of ILD is granulomatous-lymphocytic interstitial lung disease (GLILD), which is described mainly in the course of CVID with a frequency of about 8-20% (10). The rarest non-infectious complication is malignancy. The most common are lymphomas, the incidence of which is estimated at less than 10% of patients (9).

Pulmonary complications in patients with PADs not only impair their quality of life, but also contribute to higher mortality. In patients with CVID, it has been estimated that the risk of death increases twofold if there are functional or structural changes in the lungs (11). A higher incidence of extrapulmonary complications such as splenomegaly, lymphomas, autoimmunity (especially autoimmune cytopenias), has been described among patients with GLILD (10).

Lung imaging studies should be performed to detect pulmonary complications of PADs. Such examinations are sometimes repeated multiple times during a patient’s lifetime to monitor the disease. Currently, the gold standard for diagnosis is computed tomography (CT), especially high-resolution computed tomography (HRCT) (7, 12). Performing these tests is associated with patient exposure to X-rays or contrast agents. This limits their ability to perform tasks frequently. It is worth noting that increased radiosensitivity has also been demonstrated in patients with CVID compared with healthy individuals (13).

Lung ultrasonography (LUS) does not have these disadvantages. This method allows non-invasive diagnosis of the pleural cavities, pleura, and lungs. A disadvantage of LUS, and a prerequisite for imaging pulmonary lesions, is that they are in direct contact with the pleural line. It is essential that the examination is performed at the patient’s bedside. This could be helpful when pulmonary imaging monitoring is necessary. The patient does not require any preparation, and the procedure can be repeated many times, even at short intervals.

Ultrasonography of the lungs has been an underestimated diagnostic method for many years. An appropriate air lung is a barrier to the propagation of ultrasonic waves. Initially, ultrasound was used for years only for the diagnosis of pathological lesions in the pleural cavity (fluid, neoplastic lesions of the chest wall) (14). As a result of lung disease, there is a loss of aeration (total or partial), and lesions appear on ultrasound, which are referred to as artifacts or consolidations. The artifacts do not correspond to anatomical structures but are formed when lung aeration is reduced (15). Artifacts are often accompanied by pleural lines (on the lung surface) and subpleural lesions. The constellation of individual artifacts, pleural lines, and subpleural lesions facilitates the differentiation of infectious interstitial lesions, cardiogenic pulmonary edema, and pulmonary fibrosis. The second type of lesion is a consolidation, that is, area of airless lung. Other ultrasound symptoms coexist with consolidations, allowing for further differential diagnosis of inflammatory changes such as atelectasis, infarction in the course of pulmonary embolism, and metastatic changes or abscesses (16).

The last few decades have seen an increase in the number of original publications that show promise for the use of ultrasonography in the imaging diagnosis of pulmonary lesions. To date, well-developed criteria include lesions in which there is consolidation of the pulmonary parenchyma (pneumonia, atelectasis, lesions in the course of pulmonary embolism) and interstitial lesions (cardiogenic pulmonary edema, interstitial pneumonia, pulmonary fibrosis in the course of interstitial lung disease) (17–20). The results of numerous studies make it possible to consider LUS as a useful method for the diagnosis of lung lesions in examinations using X-rays (19, 20). Lung US is particularly well-established for lower respiratory tract infections in children (21, 22).

Despite numerous possible pulmonary complications in the course of IEI, we did not find data on the ultrasound images of the lungs in this group of patients in the available literature. The aim of our study was to characterize the lesions in the lungs that can be visualized using LUS in a group of patients with PADs. An additional goal was to compare the lung images obtained using ultrasound with those obtained using high-resolution tomography.

## Material and methods

### Study group

Twenty-nine patients (13 women and 16 men) with PADs (23 with CVID, 2 with XLA, 2 with IgG subclass deficiencies, and 2 with unspecified hypogammaglobulinemia) were included in this study. The mean age at the onset of the first symptoms was 23.6 ± 13.6 years. The mean diagnosis delay was 8.0 ± 9.0 years. At the time of testing, the mean age, was 39.0 ± 11.9 years. The mean time between the first symptoms of PADs and the examination was 15.4 ± 10.1 years. All patients received immunoglobulin replacement therapy (3 IVIG and 26 SCIG/fSCIG). The mean IgG concentration at the time of lung imaging studies was 8.4 ± 1.8 g/l. Most patients (26/29, 89.7%) declared that they had a history of recurrent lower respiratory tract infections. Twelve patients had infections only until the diagnosis of PADs was made and immunoglobulin replacement therapy introduced. While imaging studies were performed, we did not observe any clinical signs of respiratory tract infections. The most common noninfectious complication was polyclonal lymphoproliferation (n=16; 55.2%). followed by 51.7% autoimmunity, 44.8% pulmonary fibrosis, 34.5% bronchiectasis, 17.2% GLILD and 13.8% asthma. A detailed characteristic of the population is available in the **Supplementary Material**.

The inclusion criteria were as follows: age ≥18 years, diagnosis of PADs according to the diagnostic criteria of the European Society for Immunodeficiencies (23), and provision of written consent. The exclusion criteria were as follows: unfulfilled inclusion criteria, symptoms of acute respiratory tract infection, and in the case of women, pregnancy.

### Lung ultrasound

**Figure 1:**
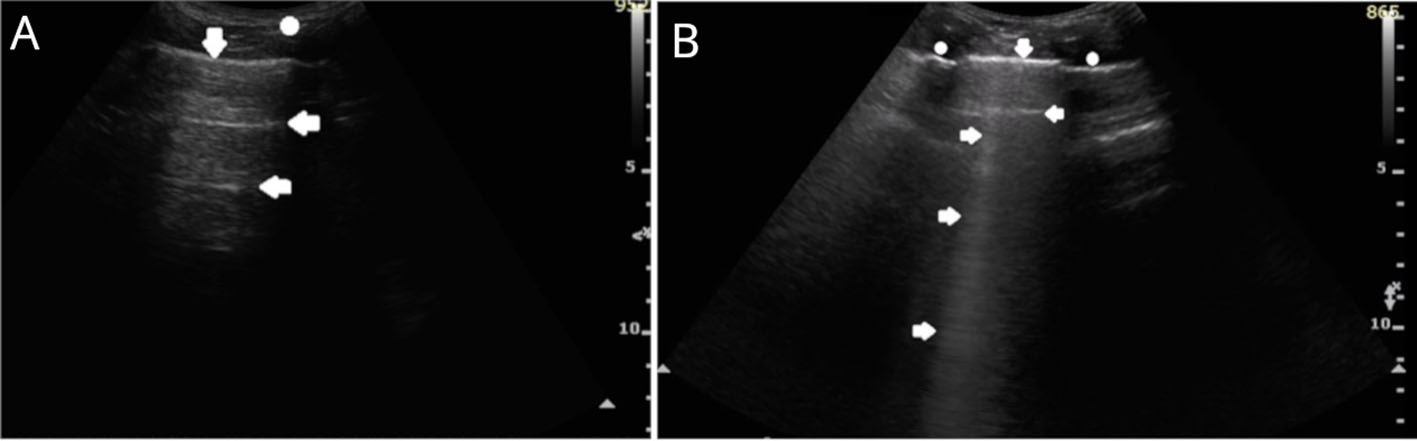
Image of normal lung on ultrasonography. **(A)** structures of chest wall (o), smooth and regular pleural line (↓), A lines, horizontal artifacts observed in properly aerated lung (←). Convex probe (1-6MHz). **(B)** ribs and anechoic shadow behind them (o), smooth and regular pleural line (↓), A line artifact (←), B line, vertical artifact of comet tail, in some objects visible in the last intercostal space as a normal variant (→). Convex probe (1-6MHz).

Lung ultrasound was performed with a PHILIPS ultrasound scanner (year of manufacture 2016, WA, USA) using two probes: convex (2–6 MHz) and linear (4–12 MHz). Ultrasound examination of the lungs was performed according to a protocol involving scanning the entire lung surface available during the ultrasound examination bilaterally over the posterior, lateral, and anterior chest wall. We presented an ultrasound image of a normal lung in **Figure 1**.

**Figure 2:**
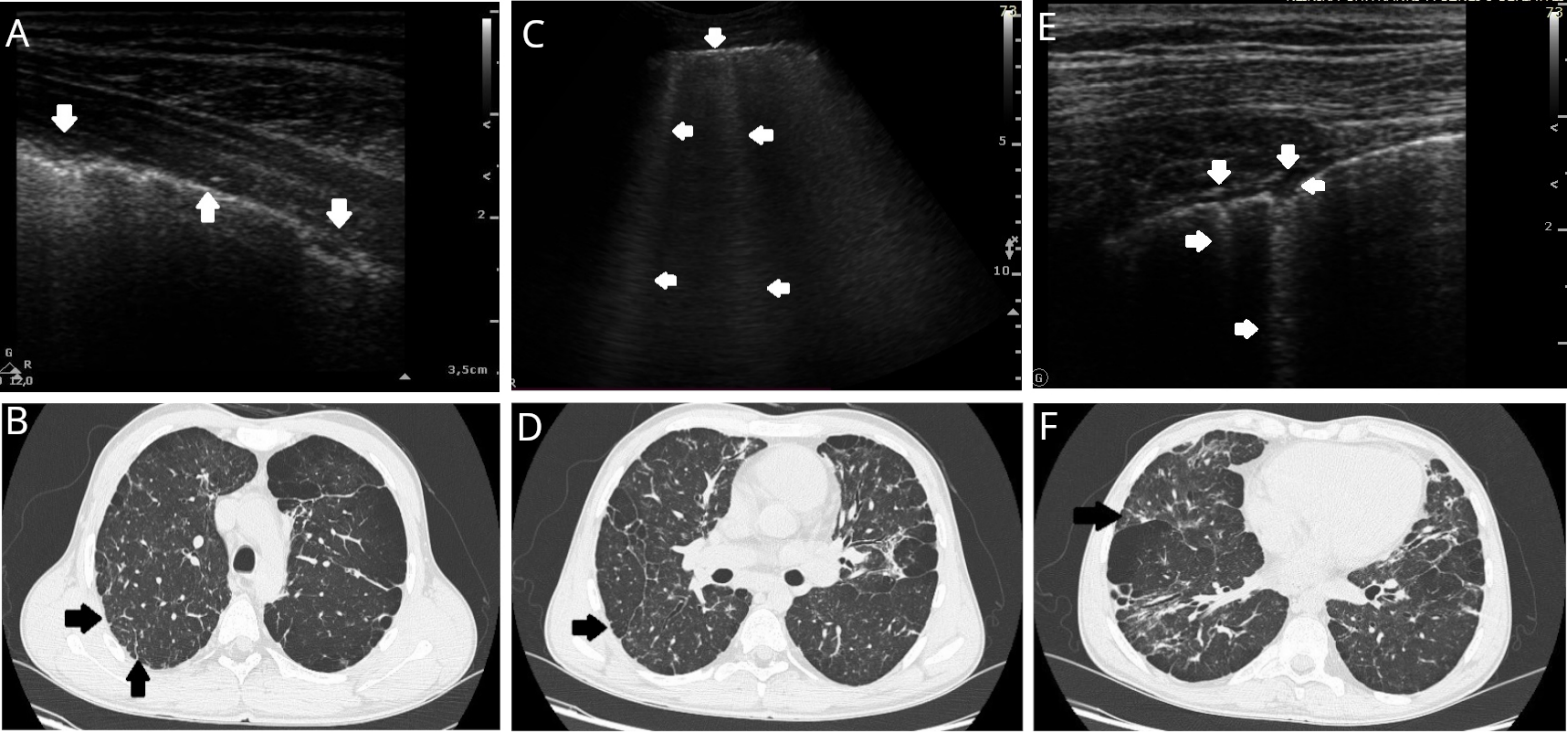
Examples of lung lesions in a patient with predominantly antibody deficiency on lung ultrasound (LUS) and high-resolution tomography (HRCT). **(A)** LUS: Irregular and infiltrated pleural line (↓), regular pleural line (↑). Linear probe. **(B)** HRCT (axial plane): Right lung - peripheral paraseptal emphysema with centrilobular emphysema inside the lung and fibrosis, thickening of the pleura between the arrows with subpleural fibrosis (arrowheads). Left lung combined centrilobular and panlobular emphysema with fibrosis. **(C)** LUS: Irregular pleural line (↓) and multiple B lines (←). Convex probe (1-6MHz). **(D)** HRCT (axial plane): Right lung - peripheral paraseptal emphysema with centrilobular emphysema inside the lung and fibrosis (arrowhead). Left lung combined paraseptal and centrilobular emphysema with fibrosis. **(E)** LUS: Irregular pleural line, and small subpleural consolidation (↓←) with vertical artifact C line (arising from subpleural lesion) (→). Linear probe. **(F)** HRCT (axial plane): Right lung – combined paraseptal, centrilobular and panlobular emphysema and fibrosis, thickening of the pleura with subpleural fibrosis (arrowhead). Left lung combined paraseptal, centrilobular and panlobular emphysema with fibrosis.

The lesions observed in each lung field were anonymized in a dedicated form and submitted for statistical analysis. Ultrasound examinations were performed by a lung ultrasound specialist with 12 years of experience. Pulmonary fibrosis was assumed to be present in a region if the following criteria were met: pleural lesions (irregularity, fragmentation, blurred pleural line), vertical artifacts (B lines, Z lines, C lines), and subpleural consolidations. In **Figure 2**, we have shown examples of pathological changes in lung ultrasound images.

### Chest high-resolution tomography

Chest HRCT scans were obtained using a 128-detector row Siemens Somatom Flash scanner (Siemens, Forchheim, Germany). Images were obtained in the craniocaudal direction during a single breath-hold with collimation 128 × 0.6 mm, rotation time 0.5 s, matrix 512 × 512 mm, and 0.6 mm reconstructed section thickness. Image analysis was performed using dedicated software (Syngo.via, Siemens) and an application (CT Chest in Syngo.via) with standard lung window settings (width,−50 HU; level, 1500 HU) and mediastinal window settings (width, 350 HU; level, 50 HU). HRCT was performed within 2 hours of lung ultrasound.

The lesions observed on HRCT were described in detail in a form dedicated to CT lesion descriptions and anonymized for further statistical analysis. Computed tomographic scans were reviewed by a radiologist with 18 years of experience. In both examinations, lesions were compared in the same 12 regions: for each lung in the upper, middle, and lower parts, separately, front and back.

### Statistical methods

Statistical analyses were performed using the STATISTICA software (version 13; TIBCO Software Inc., Palo Alto, CA, USA). To determine the relationship between abnormalities detected on HRCT and LUS, a chi-square test or Fisher’s exact test (when the expected number was smaller than five) was employed with the phi coefficient as a measure of the power of correlation. Statistical significance was assumed at p < 0.05.

### Bioethics committee

Studies involving human participants were reviewed and approved by the Ethics Committee of the Medical University of Gdansk, Gdańsk, Poland. All participants provided written informed consent to participate in the study.

## Results

### Lung ultrasonography

We described 435 lesions on lung ultrasonography in our study group of 29 patients with predominantly antibody deficiency. Most lesions were located in the lower regions of the lungs (265; 60.9%). The numbers of lesions in the middle and upper parts were 115 (26.4%) and 55 (12.7%), respectively. The most frequently described lesion was consolidation (n = 99; 22.8%). The frequencies of other lesions were as follows: C-lines (94, 21.6%), irregular pleural lines (93, 21.4%), B-lines (57, 13.1%), fragmented pleural line (45, 10.3%), blurred pleural lines (24, 5.5%), and Z-lines (23, 5.3%). **Table 1** shows the number of lesions described in each of the 12 lung regions examined in this study. Pulmonary fibrosis, diagnosed according to the established definition, was diagnosed 79 times. Fibrosis was most common in the lower lung (48; 60.8%). In the middle and upper regions of the lungs, 20 (25.3%) and 11 (13.9%) lesions were suggestive of fibrosis, respectively (**Table 2**). In five patients (17.2%), no pathological lesions were detected on ultrasound.

**Table 1:** Distribution of each lesion detected in lung ultrasound (LUS) and high-resolution computed tomography (HRCT) by lung region.

	LUNG ULTRASONOGRAPHY					HIGH-RESOLUTION COMPUTED TOMOGRAPHY				
	Lesions	FRONT		BACK		Lesions	FRONT		BACK	
		LEFT	RIGHT	LEFT	RIGHT		LEFT	RIGHT	LEFT	RIGHT
**TOP**						Fibrosis	1 (3.4%)	5 (17.2%)	2 (6.9%)	5 (17.2%)
	Blurred PL	1 (3.4%)	0 (0.0%)	0 (0.0%)	0 (0.0%)	PL thickening	0 (0.0%)	0 (0.0%)	2 (6.9%)	1 (3.4%)
	Irregular PL	2 (6.9%)	1 (3.4%)	2 (6.9%)	4 (13.8%)	Calcifications	0 (0.0%)	0 (0.0%)	1 (3.4%)	0 (0.0%)
	Fragmented PL	1 (3.4%)	0 (0.0%)	0 (0.0%)	3 (10.3%)	PL adhesions and clusters	0 (0.0%)	0 (0.0%)	0 (0.0%)	1 (3.4%)
	B-lines	2 (6.9%)	3 (10.3%)	3 (10.3%)	0 (0.0%)	Bronchiectasis	0 (0.0%)	0 (0.0%)	0 (0.0%)	1 (3.4%)
	C-lines	4 (13.8%)	4 (13.8%)	2 (6.9%)	4 (13.8%)	Emphysema bulls	0 (0.0%)	1 (3.4%)	0 (0.0%)	1 (3.4%)
	Z-lines	1 (3.4%)	2 (6.9%)	1 (3.4%)	1 (3.4%)	Thickening of the ILS	0 (0.0%)	0 (0.0%)	1 (3.4%)	1 (3.4%)
	Consolidations	4 (13.8%)	4 (13.8%)	2 (6.9%)	4 (13.8%)	Consolidations	0 (0.0%)	0 (0.0%)	0 (0.0%)	0 (0.0%)
						Tree-in-bud	0 (0.0%)	1 (3.4%)	0 (0.0%)	1 (3.4%)
**MIDDLE**						Fibrosis	4 (13.8%)	6 (20.7%)	5 (17.2%)	8 (27.6%)
	Blurred PL	1 (3.4%)	1 (3.4%)	0 (0.0%)	1 (3.4%)	PL thickening	1 (3.4%)	0 (0.0%)	5 (17.2%)	5 (17.2%)
	Irregular PL	5 (17.2%)	7 (24.1%)	6 (20.7%)	6 (20.7%)	Calcifications	0 (0.0%)	0 (0.0%)	1 (3.4%)	1 (3.4%)
	Fragmented PL	3 (10.3%)	4 (13.8%)	3 (10.3%)	3 (10.3%)	PL adhesions and clusters	2 (6.9%)	0 (0.0%)	0 (0.0%)	0 (0.0%)
	B-lines	2 (6.9%)	2 (6.9%)	5 (17.2%)	6 (20.7%)	Bronchiectasis	1 (3.4%)	2 (6.9%)	1 (3.4%)	2 (6.9%)
	C-lines	9 (31.0%)	10 (34.5%)	2 (6.9%)	5 (17.2%)	Emphysema bulls	1 (3.4%)	0 (0.0%)	1 (3.4%)	1 (3.4%)
	Z-lines	1 (3.4%)	3 (10.3%)	2 (6.9%)	1 (3.4%)	Thickening of the ILS	1 (3.4%)	3 (10.3%)	1 (3.4%)	2 (6.9%)
	Consolidations	9 (31.0%)	10 (34.5%)	3 (10.3%)	5 (17.2%)	Consolidations	0 (0.0%)	1 (3.4%)	0 (0.0%)	3 (10.3%)
						Tree-in-bud	1 (3.4%)	2 (6.9%)	3 (10.3%)	4 (13.8%)
**BOTTOM**						Fibrosis	8 (27.6%)	9 (31.0%)	13 (44.8%)	8 (27.6%)
	Blurred PL	6 (20.7%)	5 (17.2%)	5 (17.2%)	4 (13.8%)	PL thickening	0 (0.0%)	1 (3.4%)	4 (13.8%)	4 (13.8%)
	Irregular PL	15 (51.7%)	15 (51.7%)	14 (48.3%)	16 (55.2%)	Calcifications	1 (3.4%)	2 (6.9%)	0 (0.0%)	1 (3.4%)
	Fragmented PL	6 (20.7%)	8 (27.6%)	8 (27.6%)	6 (20.7%)	PL adhesions and clusters	7 (24.1%)	5 (17.2%)	5 (17.2%)	3 (10.3%)
	B-lines	5 (17.2%)	9 (31.0%)	11 (37.9%)	9 (31.0%)	Bronchiectasis	3 (10.3%)	4 (13.8%)	6 (20.7%)	2 (6.9%)
	C-lines	15 (51.7%)	16 (55.2%)	10 (34.5%)	13 (44.8%)	Emphysema bulls	1 (3.4%)	1 (3.4%)	1 (3.4%)	1 (3.4%)
	Z-lines	5 (17.2%)	4 (13.8%)	0 (0.0%)	2 (6.9%)	Thickening of the ILS	1 (3.4%)	2 (6.9%)	3 (10.3%)	1 (3.4%)
	Consolidations	15 (51.7%)	16 (55.2%)	14 (48.3%)	13 (44.8%)	Consolidations	3 (10.3%)	1 (3.4%)	0 (0.0%)	0 (0.0%)
						Tree-in-bud	3 (10.3%)	4 (13.8%)	5 (17.2%)	3 (10.3%)

PL, pleural line; ILS, interlobular septum. Frequency is shown using a color scale from lowest (green) to highest (red) separately for LUS and HRCT.

**Table 2:** Analysis of the frequency of fibrosis with the coefficient phi for the correlations between findings detected in lung ultrasound and high-resolution computed tomography.

	FRONT										BACK									
	LEFT					RIGHT					LEFT					RIGHT				
**TOP**	**USG**	**HRCT**				**USG**	**HRCT**				**USG**	**HRCT**				**USG**	**HRCT**			
		**No**		**Yes**			**No**		**Yes**			**No**		**Yes**			**No**		**Yes**	
		**N**	**%**	**N**	**%**		**N**	**%**	**N**	**%**		**N**	**%**	**N**	**%**		**N**	**%**	**N**	**%**
	**No**	26	100.0 %	0	0.0 %	**No**	24	100.0 %	1	20.0 %	**No**	27	100.0 %	0	0.0 %	**No**	24	100.0 %	3	60.0 %
	**Yes**	0	0.0 %	3	100.0 %	**Yes**	0	0.0 %	4	80.0 %	**Yes**	0	0.0 %	2	100.0 %	**Yes**	0	0.0 %	2	40.0 %
		**p**	< 0.001	**Phi**	1.00		**p**	<0.001	**Phi**	0.876		**p**	0.002	**Phi**	1.00		**p**	0.025	**Phi**	0.596
**MIDDLE**	**USG**	**HRCT**				**USG**	**HRCT**				**USG**	**HRCT**				**USG**	**HRCT**			
		**No**		**Yes**			**No**		**Yes**			**No**		**Yes**			**No**		**Yes**	
		**N**	**%**	**N**	**%**		**N**	**%**	**N**	**%**		**N**	**%**	**N**	**%**		**N**	**%**	**N**	**%**
	**No**	22	100.0 %	4	57.1 %	**No**	22	95.7 %	0	0.0 %	**No**	22	100.0 %	2	28.6 %	**No**	17	94.4 %	7	63.6 %
	**Yes**	0	0.0 %	3	42.9 %	**Yes**	1	4.3 %	6	100.0 %	**Yes**	0	0.0 %	5	71.4 %	**Yes**	1	5.6 %	4	36.4 %
		**p**	0.010	**Phi**	0.602		**p**	< 0.001	**Phi**	0.905		**p**	< 0.001	**Phi**	0.809		**p**	0.054	**Phi**	0.396
**BOTTOM**	**USG**	**HRCT**				**USG**	**HRCT**				**USG**	**HRCT**				**USG**	**HRCT**			
		**No**		**Yes**			**No**		**Yes**			**No**		**Yes**			**No**		**Yes**	
		**N**	**%**	**N**	**%**		**N**	**%**	**N**	**%**		**N**	**%**	**N**	**%**		**N**	**%**	**N**	**%**
	**No**	13	86.7 %	6	42.9 %	**No**	11	78.6 %	4	26.7 %	**No**	10	83.3 %	6	35.3 %	**No**	13	86.7 %	5	35.7 %
	**Yes**	2	13.3 %	8	57.1 %	**Yes**	3	21.4 %	11	73.3 %	**Yes**	2	16.7 %	11	64.7 %	**Yes**	2	13.3 %	9	64.3 %
		**p**	0.021	**Phi**	0.461		**p**	0.009	**Phi**	0.519		**p**	0.022	**Phi**	0.476		**p**	0.008	**Phi**	0.525

### High-sensitive computed tomography

Compared with LUS, the number of lesions described on HRCT was lower, amounting to 209. However, in this study, we also described most changes in the lower parts of the lungs (116; 55.5%). There were 25 (12.0%) and 68 (32.5%) lesions in the upper and middle lung regions, respectively (**Table 1**). The most frequently described lesion was fibrosis (n = 74, 16.5%). The frequencies of other lesions were as follows: tree-in-bud pattern (27, 6.0%), pleural thickening (23, 5.1%), pleural adhesions (23, 5.1%), bronchiectasis (22, 4.9%), thickening of the intertrabecular septum (16, 3.6%), emphysema bulls (9, 2%), consolidations < 5 mm (8, 1.8%), and calcifications (7, 1.6%). In three patients (10.3%), no pathological changes were observed on HRCT. Examples of pathological changes observed on HRCT are shown in **Figure 2**.

### Comparison of pulmonary fibrosis in HRCT and LUS

We found no correlation between the occurrence of individual lesions on LUS and lesions observed on HRCT. However, it should be emphasized that the analysis of LUS results is based on the occurrence of certain combinations of symptoms rather than individual findings.

To compare the diagnostic capabilities of HRCT and LUS, we assessed the incidence of lesions indicative of pulmonary fibrosis in both studies. We chose fibrosis because it is the most frequently described lesion on computed tomography, and a set of ultrasound signs are known to identify this process in LUS. **Table 2** summarizes the prevalence of fibrosis in the 12 examined regions of the lungs. A statistically significant relationship between the results of both imaging studies was found in 11 regions. The strength of the relationship was strong (phi = 0.40–0.69) or very strong (phi ≥ 0.70). The maximum values of the phi coefficient for the upper part of the left lung were recorded (phi = 1.0). Only the result for the back in the middle of the right lung was at the limit of statistical significance (p = 0.054). In the same region, the strength of the association between the results of the two examinations, assessed using the phi coefficient, was the weakest (phi = 0.396).

## Discussion

Lung disease is a frequent complication of PADs with high morbidity and mortality rates. The spectrum of clinical manifestations is broad, and includes acute and chronic infections, structural abnormalities, and malignancies (3, 8). All these disorders have in common that diagnostic imaging is necessary to establish the diagnosis and monitor progression. Currently, we mainly use computed tomography for this purpose (7). In many groups of patients, the usefulness of lung ultrasound, which has been developing intensely in recent years, has been proven. To our knowledge, ultrasound lung lesions in patients with primary immunodeficiencies have not yet been described.

In our group of 29 patients with PADs, the lesions described on both LUS and HRCT were usually diffuse rather than focal. In most cases, the lesions closely resembled those described in interstitial lung disease. Twenty-four patients had multiple ultrasound abnormalities in the form of artifacts (B-, C-, and Z-line artifacts), pleural line lesions (irregular, fragmented, and blurred), and small subpleural consolidations (< 5 mm). Consolidations and accompanying pleural line lesions in the LUS were the most frequent, which may indicate lesions in the interstitial space and alveoli. These lesions may be secondary to atelectasis or post-inflammatory changes, which may be due to previous recurrent lower respiratory tract infections. It should be noted that vertical artifacts observed in large numbers upon LUS examination are an indirect parameter indicating a problem located in the interstitial space of the lungs or in the subpleural area.

The higher number of pleural lesions described on lung ultrasound than on HRCT may be due to technical differences between these examinations. LUS allows for very accurate imaging of the pleural line and superficial parts of the lungs compared to CT. If interstitial lung lesions are predominant, LUS does not allow the assessment of deeper lung areas. On the other hand, computed tomography allows deep evaluation of the lung up to the mediastinum. Lung ultrasound and HRCT are complementary and used together may allow for improved diagnostic and monitoring capabilities for patients with PADs.

In our group, both LUS and HRCT showed that the lesions accumulated mainly in the lower and middle parts of the lungs. These observations are consistent with previously published lesion locations on HRCT in patients with PADs. Both Tanaka et al. (24) and Bondionii et al. (25) observed very few lesions in adult patients with CVID and XLA in the upper lung on HRCT. They were predominantly in the middle and lower parts. However, the accumulation of lesions in the lower lobes of the lungs, as observed in our study, has not been described.

In the CT scan performed up to 2 h after the LUS, numerous non-specific abnormalities were found in the studied patients. We found no correlation between individual lesions on LUS examination and lesions observed on HRCT. This is due to the fact that in LUS, it is not individual lesions but their co-occurrence in certain constellations that should be evaluated. This makes it impossible to directly compare the deviations described by the lung imaging techniques.

The most common lesions on HRCT are indicative of lung fibrosis. In case of LUS, we defined the features of the ultrasound image that indicated the presence of this pathology. We demonstrated a statistically significant and strong correlation between fibrotic images on LUS and HRCT. This supports the usefulness of ultrasonography in the diagnosis of pulmonary fibrosis, which is also described for idiopathic pulmonary fibrosis (26) or lesions in the course of systemic connective tissue diseases (19).

In the study group, no patient was found to have neoplastic disease; therefore, it is not possible to conclude on the diagnostic possibilities of neoplastic disease with lung ultrasonography.

Surprisingly, there were a low number of bronchiectasis cases in the study group. These lesions accounted for approximately 5% of all lesions described on HRCT. Bronchiectasis is a common complication of CVID. In the study group of patients with PADs, the majority had this immunodeficiency. According to various estimates, the percentage of patients with CVID diagnosed with bronchiectasis ranges from 25 to 79% (7). The low incidence of this complication in our group may have been due to well-managed immunoglobulin supplementation. Indeed, a close relationship between the incidence of bronchiectasis and IgG levels has been previously demonstrated (27).

Our study has a few limitations. We included a small group of patients; however, this population was well clinically characterized. This was a pilot study, and we performed the examinations only once. We did not analyze how the lung lesions changed during a longer follow-up period. Owing to the very high variability of the described abnormalities in both imaging studies and the different clinical presentations of PADs, it is necessary to conduct studies on a larger number of patients. To compare the usefulness of LUS and HRCT, it would be worthwhile to conduct a study on a group of patients with well-defined pulmonary complications. This will allow for a comparison of the two imaging studies in specific clinical situations. In future studies it would be worthwhile to correlate functional test results with lung ultrasound images.

## Conclusions

In the study group of patients with predominantly antibody deficiencies, the diagnostic potential of ultrasonography for the evaluation of pulmonary lesions was evaluated. The lesions on LUS and HRCT were non-specific. The features of fibrosis found by both diagnostic methods correlated very well. Lung ultrasonography appears to be a promising method for imaging pulmonary lesions, especially fibrosis, in patients with primary immunodeficiencies. For lesions of a different nature, it is necessary to perform studies on a larger group of patients with strictly defined pulmonary complications.

## Data availability statement

The raw data supporting the conclusions of this article will be made available by the authors, without undue reservation.

## Ethics statement

The studies involving human participants were reviewed and approved by Ethics Committee of the Medical University of Gdansk, Gdańsk, Poland. The patients/participants provided their written informed consent to participate in this study.

## Author contributions

MZ and NB designed the study and wrote the first draft of the manuscript. This text was produced with equal contributions from both authors. MZ, NB, EW-S, and DG collected the data and performed the literature searches. NB performed the lung ultrasound examinations. MP described HRCT findings. MZ performed the statistical analyses. EW-S, ZZ, and KJ-R critically revised the manuscript for intellectual content. All the authors have read and agreed to the published version of the manuscript.

## Conflict of interest

The authors declare that the research was conducted in the absence of any commercial or financial relationships that could be construed as potential conflicts of interest.

## Publisher’s note

All claims expressed in this article are solely those of the authors and do not necessarily represent those of their affiliated organizations, or those of the publisher, the editors and the reviewers. Any product that may be evaluated in this article, or claim that may be made by its manufacturer, is not guaranteed or endorsed by the publisher.
